# Experimental Study on Drilling Signal Characteristics of PDC Drill Bit in Media of Different Strengths and Identification of Weak Media

**DOI:** 10.3390/s24237852

**Published:** 2024-12-08

**Authors:** Zheng Wu, Yingbo Fan, Huazhou Chen

**Affiliations:** 1 Institute of Remote Sensing and Geographic Information System, Peking University, Beijing 100871, China; ybfan@stu.pku.edu.cn; 2 Institute of Energy, School of Earth and Space Sciences, Peking University, Beijing 100871, China; chenhuazhou@stu.pku.edu.cn

**Keywords:** measurement while drilling, roadway support, roof collapse hazard identification, time–frequency analysis

## Abstract

This study aimed to investigate the drilling signal characteristics when a PDC drill bit penetrates media of different strengths and to assess the potential of these signals for identifying weak layers within rock formations. Laboratory-scale experiments were conducted, and the response characteristics of the PDC drill bit in different-strength media were analyzed across the time domain, frequency domain, and time–frequency domain using statistical analysis, Fourier transform, and empirical mode decomposition (EMD). The results indicate that in the lowest-strength concrete (C10), the drilling speed was the fastest, while the mean, median, and primary distribution ranges of the thrust and torque were the smallest. Some dimensionless time-domain and frequency-domain indicators were found to have limitations in differentiating media of varying strengths. Meanwhile, the time–frequency analysis and EMD of the thrust and torque signals revealed distinct changes at the media boundaries, serving as auxiliary criteria for identifying transitions between different media. The time–frequency analysis and EMD demonstrated clear advantages in identifying these boundaries. These findings provide a theoretical basis for using drilling signals to identify weak layers that pose potential roof collapse hazards in roadway roof strata.

## 1. Introduction

Roof hazards pose significant threats in coal mine production due to their concealed nature. Small joints, fractures, and minor structures within coal-bearing, stratified formations are difficult to fully detect and identify in the early stages. Under the influence of mining-induced stress, weak planes within these rock layers are prone to failure, leading to the local instability of the roadway roof and potentially causing collapse accidents. The construction of the roof bolt holes is a necessary and crucial process in roadway excavation operations. By integrating the installation of roof bolts and cables with real-time drilling signal analysis, and thoroughly analyzing and utilizing the signal data obtained during drilling, a more detailed supplementary exploration of the roof rock conditions in the roadway area can be conducted. The timely identification and detection of the rock layer conditions within the drilling range, as well as the potential roof collapse hazard zones during drilling operations, are of great significance and have promising application prospects for enhancing roadway safety and reducing the occurrence of roof collapse incidents.

In this study, we employed laboratory-scale experiments to comprehensively analyze the time-domain and time–frequency characteristics of the drilling signals when a mining two-wing PDC drill bit penetrated different-strength specimens. Statistical methods, Fourier transform, and EMD techniques were applied in this analysis.

The main contributions are as follows:

(1) A multi-parameter, multi-indicator analysis method for drilling signals was proposed. From the perspectives of the time domain, frequency domain, and time–frequency domain, we applied methods such as statistical analysis, Fourier transform analysis, and EMD to conduct an in-depth analysis of the drilling signals when a PDC drill bit penetrates concrete media of varying strengths. The effectiveness of the indicators, like the average drilling speed and the time–frequency characteristics of the torque signals, was verified by reflecting the strength of the drilled media and identifying the interfaces between different media;
(2) The limitations of the characterization of the drill-following signals were analyzed and the direction of improvement was proposed. It was discovered that the dimensionless time-domain indicators and frequency-domain features of the drilling signals are insufficient for distinguishing weak media. Specifically, some indicators—such as the pulse factor, peak factor, and margin factor—are ineffective at accurately differentiating drilled media of varying strengths. This finding provides guidance for subsequent enhancements in signal analysis methods to improve the precision of media identification.

The remainder of this paper is organized as follows: In Section 2, we review related work by other researchers. Section 3 describes the experimental design of the laboratory-scale drilling experiments. In Section 4, we conduct statistical analysis, time-domain and time–frequency analyses, and EMD of the drilling signals from the laboratory experiments. Section 5 discusses the experimental results.

## 2. Related Work

Vibration signals are vital for extracting insights during drilling. Han [1] collected vibration signals from drill bits and analyzed features like the variance and energy, effectively assessing rock layers during different operational states. Liu [2] analyzed drill string vibration signals in the time and frequency domains, extracting feature vectors and applying dimensionality reduction and reorganization. These features effectively characterized the drilling conditions and rock formations. Yue [3] found the drilling speed to be the most sensitive parameter for identifying rock interface changes, with the drilling pressure also impactful, but the rotational speed showed minimal sensitivity. Gao [4] proposed a method for predicting the Uniaxial Compressive Strength (UCS) using digital core drilling, providing a basis for continuous and rapid rock strength detection. Deng [5] simulated drilling processes across rock types, revealing that the torque and thrust correlate positively with the UCS, while the drilling speed inversely correlates, providing insights into rock characteristics. Zhang [6] designed a micro-drilling test platform to classify rocks based on while-drilling parameters. Stehlikova [7] applied machine learning to vibration signal data for rock type classification during rotary drilling, emphasizing energy optimization. Zhang [8] achieved a 98.79% accuracy in rock–coal interface identification using stacked autoencoders and acceleration sensors, showing strong application potential. Wang [9] utilized high-frequency sensors to record drill string vibrations and trained neural networks to classify rock layers with an 89.57% average accuracy, effectively distinguishing various rock types, such as soil and mudstone. Flegner [10,11] employed vibration acoustic signals and clustering to classify the drilling conditions and optimize the performance, highlighting key signal symptoms like variability and kurtosis to inform the process adjustments. Ding [12] developed a method using wavelet scattering transform and BiLSTM to classify coal–rock structures with 100% accuracy, advancing automated and intelligent drilling operations. Qin [13] proved that broadband acoustic sensors could outperform traditional vibration sensors, providing better signal-to-noise ratios and more distinct time–frequency characteristics for accurate lithology recognition. Khoshouei [14] leveraged acoustic and vibration signals to predict rock properties (e.g., UCS, BTS) with high reliability through multivariate regression. Liu [15] demonstrated that BP neural networks can use vibration signals for lithology recognition, differentiating between formations and aiding anti-collision monitoring. Gao [16] proposed using vibration response features and an optimized BP neural network for rock mass strength detection, achieving 93.75% accuracy, surpassing conventional methods.

Chang [17] used the mechanical specific energy from simulations and neural networks to identify coal–rock interfaces, providing an innovative approach to subsurface analysis. Chen [18,19] modeled drilling dynamics and analyzed vibration relationships with the rock properties, showing characteristic peak frequencies associated with different rock strengths. Jing [20] enhanced the rock-breaking efficiency with a rotary impact drilling device, improving PDC bit penetration in deep wells. Saksala [21] conducted numerical and experimental studies on impact drilling using a viscoplastic model, validated with tests on granite, showing accurate results even under high stress. Fu [22] improved the radial drilling performance using a direct-rotary hybrid nozzle, validated through experiments, which enhanced the rock breaking in low-yield wells. Yu [23] evaluated the rock strength with PFC2D modeling and compared the drillability metrics, finding a 10% higher accuracy with the rock drillability index. Zhang [24], Dong [25], and Fu [26] emphasized numerical and vibration analyses for optimizing the drilling efficiency and identifying rock properties. Their findings reinforced the importance of vibration signal analysis in understanding geological conditions and improving drilling outcomes.

Previous studies have primarily focused on utilizing measurement-while-drilling (MWD) signals to determine specific lithologies or rock mechanical parameters. In underground coal mines, the stratigraphic structure of roadway roofs can be influenced by depositional environments or other factors, which may lead to the thinning of stable rock layers or the thickening of weak layers. These abrupt changes in stratigraphic structures, especially the thickening of weak rock layers, greatly increase the risk of roof instability and collapse. These weak rock layers or media are often potential hazard areas for roof collapse, making their accurate identification crucial for ensuring roadway stability and the safety of workers. The existing research has demonstrated a strong correlation between MWD signals and drilled media properties, providing a solid basis for investigating the link between the signal characteristics and weak media. Building on these findings, this study integrated multiple sensors and employed a combination of time-domain, frequency-domain, and time–frequency-domain analyses. Statistical analysis, Fourier transform, and EMD were used to investigate the MWD signal responses to weak media and identify potential roof collapse hazard zones.

## 3. Design of Laboratory Drilling Experiment

### 3.1. Experimental Equipment

**Figure 1:**
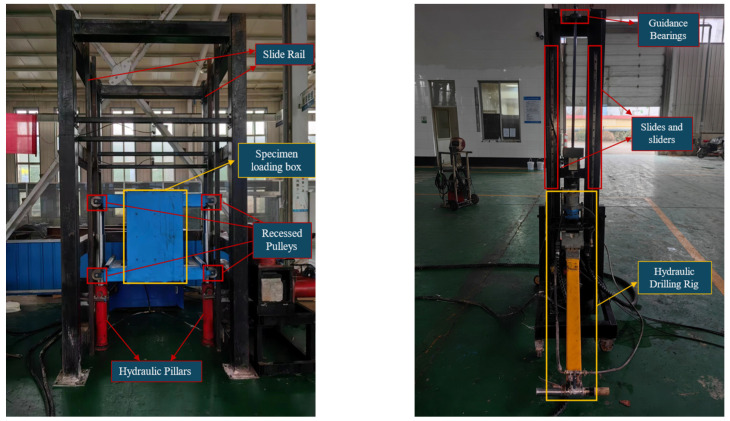
Drilling experiment platform and hydraulic anchor drilling rig.

The laboratory testing platform, shown in Figure 1, includes a specimen-loading box that accommodates multiple specimen sets. The box is raised and lowered by two hydraulic props positioned on either side, supported by grooved pulleys and slide rails. A hydraulic anchor drilling rig (model MYT-140/320 I) was chosen to simulate underground roof bolt hole construction conditions. The drilling rig is fixed onto a support frame equipped with a slideway and slider along the drilling direction. A guide bearing is installed at the top of the drill rod to ensure that the movement trajectories of the drilling rig and drill rod remain stable during drilling, avoiding significant deviations that could affect the drilling process and data acquisition.

Five types of sensors—measuring the drill thrust, drill rod rotation speed, drill rod torque, three-axis vibration velocity, and hydraulic pipeline pressure—were used to monitor the key parameters in real time during the drilling process. Additionally, a cable displacement sensor was used to indirectly obtain the drilling speed parameter of the rig.

**Figure 2:**
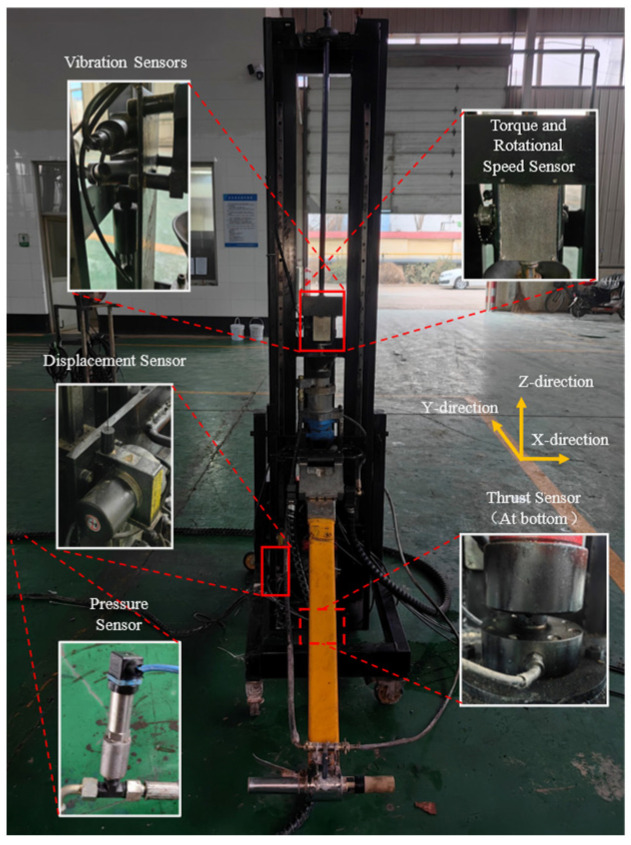
Schematic diagram of sensor installation positions.

Behind the torque sensor mounted on the drilling rig, vibration velocity sensors were installed along the drilling direction on the x, y, and z axes to capture the vibration velocities in all three directions during drilling. The definitions of the rig’s three axes are shown in Figure 2. The specifications of each sensor are listed in Table 1.

### 3.2. Drilling Experimental Design

When the structural composition of the roadway roof strata undergoes abrupt changes—such as the inclusion of weak rock layers within hard rock strata, or when the thickness of stable hard rock layers in the roof becomes thinner or even pinches out, leading to an increased thickness of weak rock layers—the overall stability of the roadway roof decreases. When the biaxial stress ratio of the surrounding rock stress field increases, failures are more likely to bypass the hard rock layers and occur within the weak rock layers. If the interbedded weak rock layer is significantly thick, its fragmentation and swelling pressure after failure can compromise the stability of the underlying rock, threatening the overall stability of the roadway roof. Additionally, if the thickness of the weak rock layer in the immediate roof increases, the distance between the roadway roof and the stable rock layer above becomes too far, making it difficult to suspend the large-scale, thick weak rock layer beneath the stable rock layer above. This makes it challenging to ensure the stability of the roadway roof using the current anchor bolt and cable support system, potentially leading to large-scale roof instability and failure.

For this type of stress-dominated roadway roof collapse, drilling experiments were designed using concrete specimens of varying strengths to accurately identify weak rock layers based on the drilling signal characteristics. Scheme 1 and Scheme 2 were used to simulate weak rock layers interbedded within relatively hard rock layers, with the height of the C10 concrete varied to represent weak layers of different thicknesses. Scheme 3 simulated an increase in the thickness of the weak rock layer in the immediate roof, resulting in a significantly greater distance between the roadway roof and the stable rock layer above.

To ensure that the properties of specimens with the same strength remained relatively uniform during the drilling experiments, and to avoid the effects of changes in the physical properties or strength parameters caused by water exposure on certain rocks, which could interfere with the drilling process and MWD signals, this study utilized concrete specimens with varying strength grades for the laboratory drilling experiments. This approach also helps mitigate the impact of unknown joints or fractures within the rock that may cause fluctuations in MWD signals and facilitate specimen transportation and handling. The lowest-strength C10 concrete specimen was used to represent weak rock layers and was designated as a potential roof collapse zone. The overall objectives of the experiment were to achieve the efficient and accurate identification of weak media by integrating multiple drilling signal characteristic indicators to validate the effectiveness and reliability of using MWD signals for identifying weak media, and to further investigate the characteristics and patterns of MWD signals in weak media.

**Figure 3:**
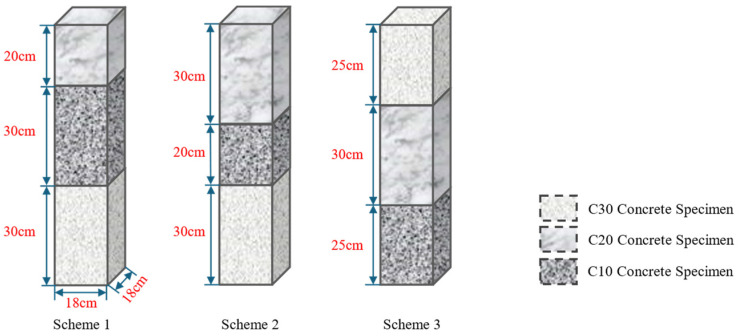
Schematic diagram of experimental setup for different-strength concrete combinations.

Drilling experiments were conducted on groups of concrete specimens with different strength grades, specifically C30, C20, and C10, in various combinations to collect drilling signals. A total of three experimental schemes were designed, as shown in Figure 3:

(1) Scheme 1: The lower section is a 30 cm C30 concrete specimen, the middle section is a 30 cm C10 concrete specimen, and the upper section is a 20 cm C20 concrete specimen, with a total height of 80 cm;
(2) Scheme 2: The lower section is a 30 cm C30 concrete specimen, the middle section is a 20 cm C10 concrete specimen, and the upper section is a 30 cm C20 concrete specimen, with a total height of 80 cm;
(3) Scheme 3: The lower section is a 25 cm C10 concrete specimen, the middle section is a 30 cm C20 concrete specimen, and the upper section is a 25 cm C30 concrete specimen, with a total height of 80 cm.

## 4. Results Analysis

### 4.1. Analysis of Drilling Signal Results for Each Experimental Group

#### 4.1.1. Experimental Results of Scheme 1

**Figure 4:**
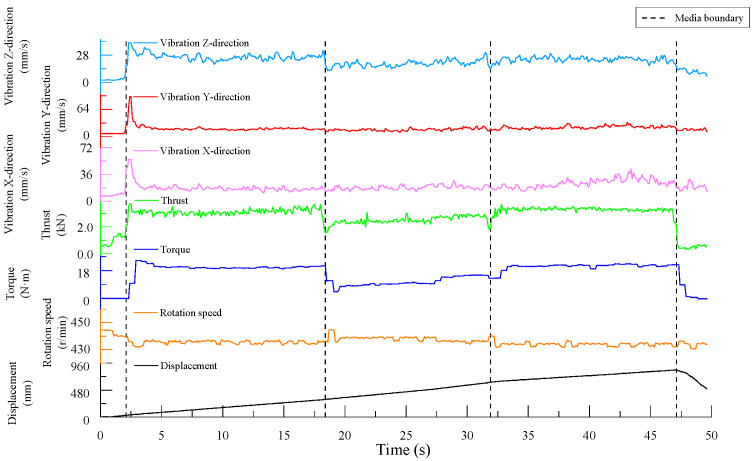
Time-domain plot of drilling signals.

As shown in Figure 4, the boundary lines in the figure divide the experimental process into five sections: before contact with the specimen, drilling into Specimen 1, drilling into Specimen 2, drilling into Specimen 3, and drill retraction after completing the drilling. Within each section, except for when the drill bit first contacted the specimen—where significant fluctuations in the drilling signals occur before a stable borehole is formed—and when nearing the end of drilling for a given specimen, where there are smaller fluctuations, the drilling signals in the middle regions remained relatively stable.

The drilling speed was the fastest in the lowest-strength C10 concrete. The thrust signal for when the drill bit reached the interface between the specimens shows a clear trend of first decreasing and then increasing, exhibiting a similar “V-shaped” pattern, and the vibration velocity in the z-direction also follows this trend. Additionally, the rotation speed shows a slight increase near the interface.

Since a stable borehole was not formed when the drill bit initially contacted the specimen, the overall vibration of the drilling rig was significant, and the data fluctuate heavily. These signals do not represent normal drilling conditions. Including these unstable signals in the statistical analysis alongside the stable signals—obtained after the drill bit establishes a consistent drilling path—would introduce considerable error in calculating the average signal data for the specimen. Therefore, only the stable drilling signals from each specimen were selected for further analysis.

Specifically, the stable signal data were taken from the following time intervals: 3.7 s to 18.1 s for the C30 concrete specimen, 19.6 s to 31.6 s for the C10 concrete specimen, and 32.8 s to 46.8 s for the C20 concrete specimen.

**Figure 5:**
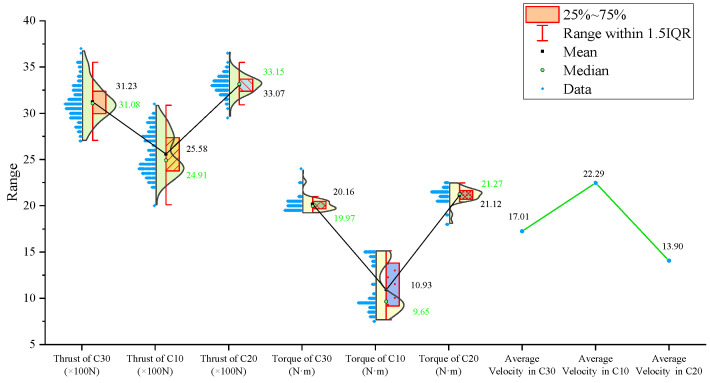
Statistical results of thrust, torque, and average drilling speed.

**Figure 6:**
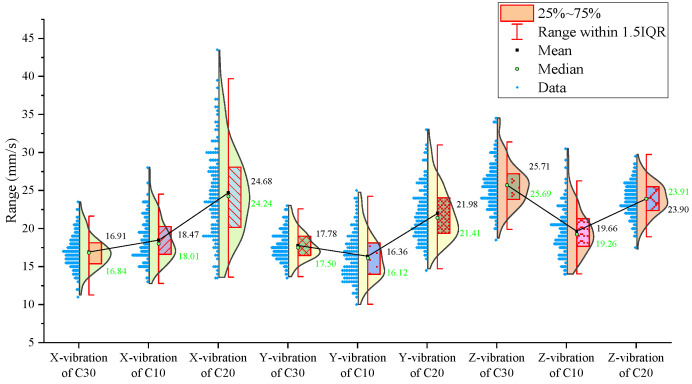
Statistical results of vibration velocities in the X, Y, and Z directions.

**Figure 7:**
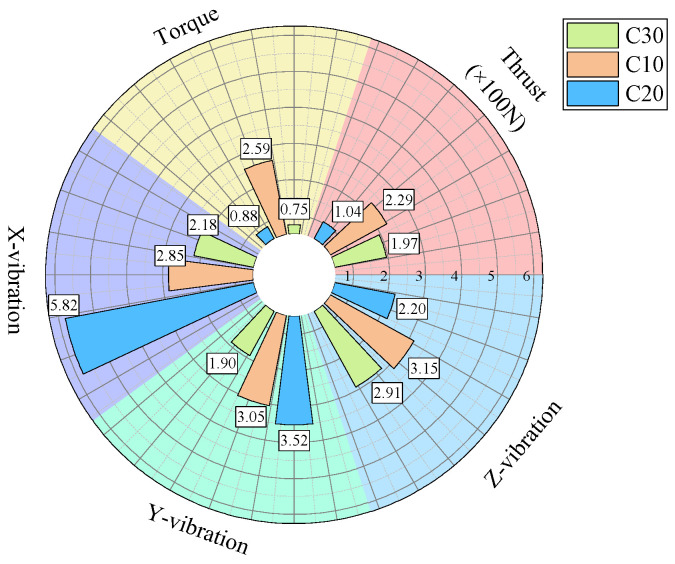
Standard deviation of drilling signals.

From the statistical graphs shown in Figure 5, Figure 6 and Figure 7, it can be observed that the C10 concrete specimen, with the lowest strength, exhibits the smallest mean, median, and primary distribution ranges for both the thrust and torque. However, the standard deviations of both the thrust and torque are the largest in the C10 concrete, indicating greater variability in these parameters during drilling.

Regarding the three-axis vibration data, both the mean and median vibration velocities in the Y and Z directions are the lowest in the C10 specimen, which has the lowest strength. Additionally, the mean vibration velocity in the Z direction shows a decreasing trend as the strength of the drilled medium increases.

#### 4.1.2. Experimental Results of Scheme Two

**Figure 8:**
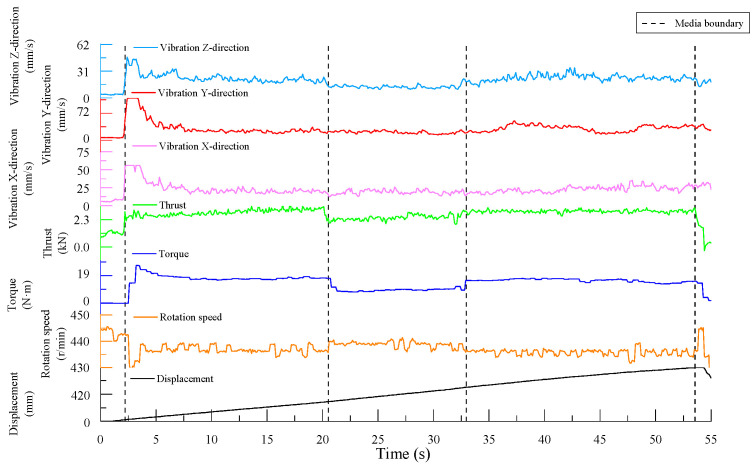
Time-domain plot of drilling signals.

The time-domain plots of the signal data are shown in Figure 8. Similar to the first set of experiments, the drilling signal data during the stable drilling phase in each specimen were selected for further analysis. Specifically, the stable signal data were taken from the following time intervals: 7.2 s to 20.1 s for the C30 concrete specimen, 21.3 s to 32.8 s for the C10 concrete specimen, and 32.9 s to 53.5 s for the C20 concrete specimen.

**Figure 9:**
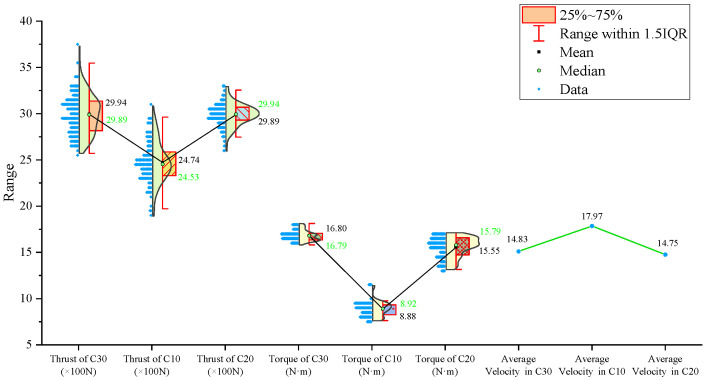
Statistical results of thrust, torque, and average drilling speed.

**Figure 10:**
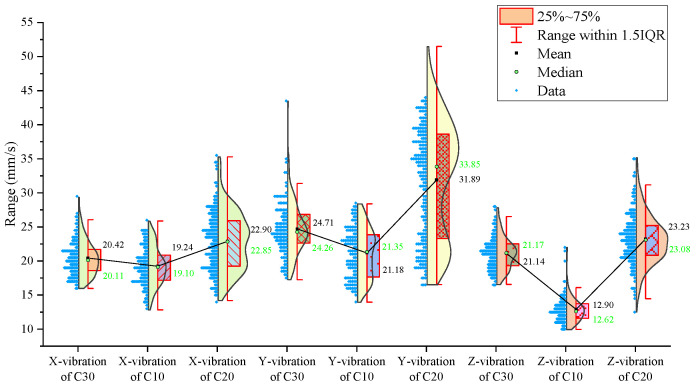
Statistical results of vibration velocities in the x-, y-, and z-directions.

**Figure 11:**
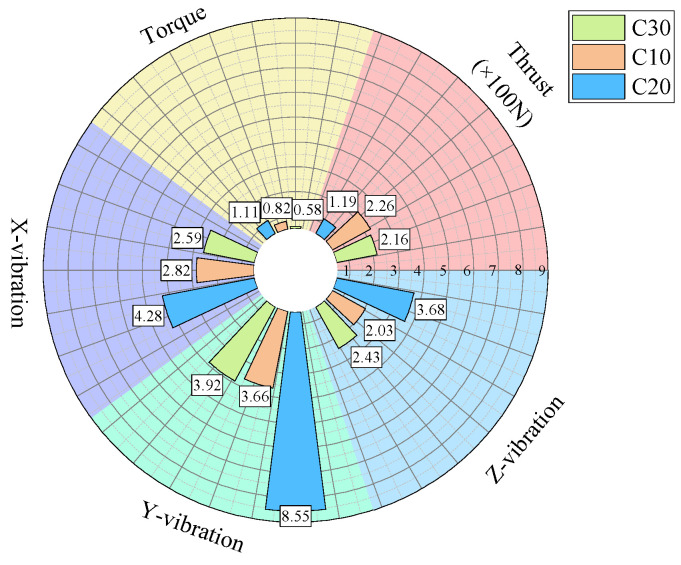
Standard deviation of drilling signals.

The signal statistics from the second set of experiments are presented in Figure 9, Figure 10 and Figure 11. As in the previous experiments, the drilling speed is the fastest in the lowest-strength C10 concrete. The mean, median, and primary distribution ranges for both the thrust and torque are the smallest for the C10 concrete. However, the standard deviation of the thrust is the largest in the C10 concrete. The mean values of both the thrust and torque exhibit an increasing trend as the strength of the drilled medium increases.

There is a slight difference in the torque standard deviation compared to the previous set of experiments: it is the smallest in the C30 concrete and the largest in the C20 concrete.

Regarding the three-axis vibration data, the mean and median vibration velocities in the x-, y-, and z-directions are the lowest in the specimen with the lowest strength (the C10 concrete). The standard deviations of the vibration velocity in the y- and z-directions are also the smallest in the C10 concrete.

#### 4.1.3. Experimental Results of Scheme Three

**Figure 12:**
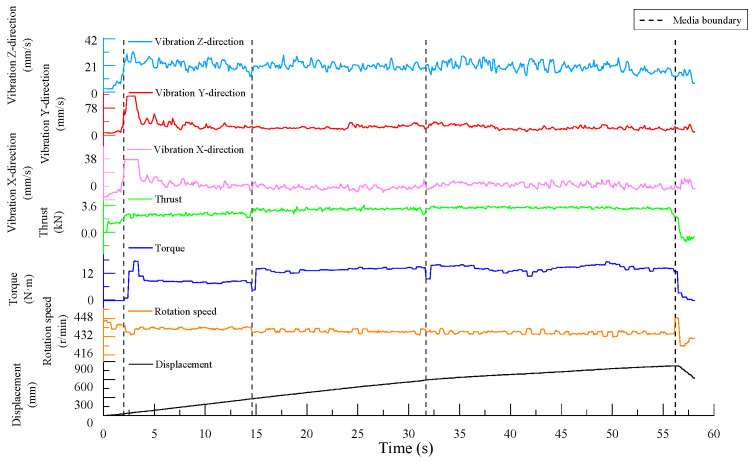
Time-domain plot of drilling signals.

The time-domain plots of the drilling signals from Experiment Group 3 are shown in Figure 12. As before, the drilling signal data from the stable drilling phases in each specimen were selected for further analysis. Specifically, the stable signal data were taken from the following time intervals: 4.5 s to 14.5 s for the C10 concrete specimen, 15.2 s to 31.6 s for the C20 concrete specimen, and 32.4 s to 55.7 s for the C30 concrete specimen.

**Figure 13:**
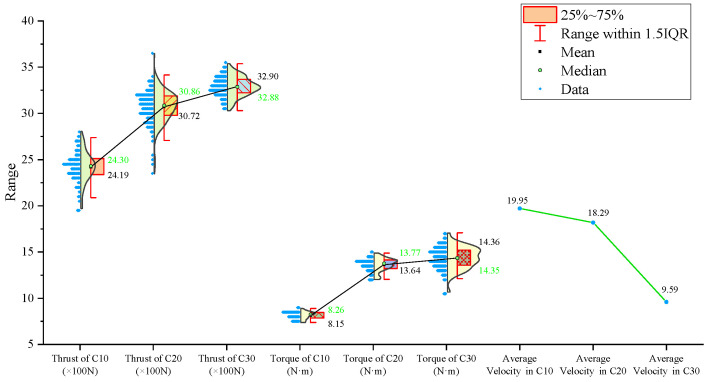
Statistical results of thrust, torque, and average drilling speed.

**Figure 14:**
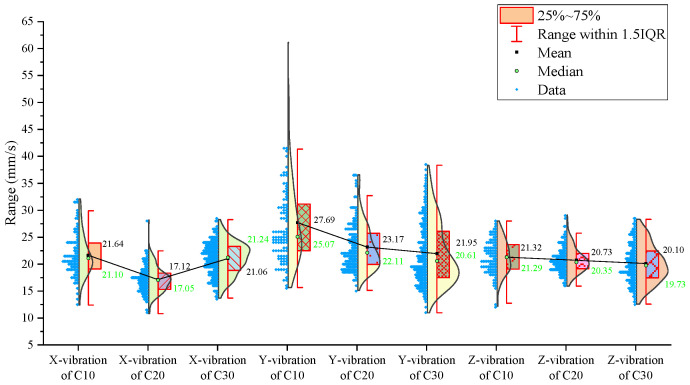
Statistical results of vibration velocities in the x-, y-, and z-directions.

**Figure 15:**
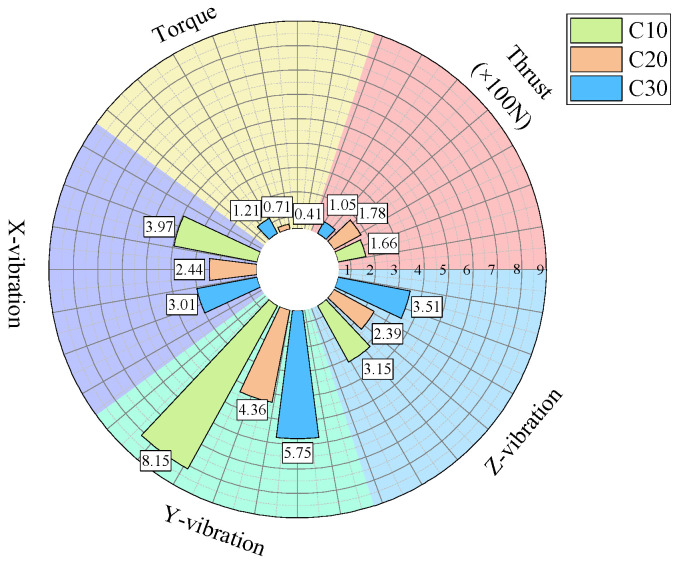
Standard deviation of drilling signals.

The signal statistics are presented in Figure 13, Figure 14 and Figure 15. Similar to the previous two experiment groups, the drilling speed in the lowest-strength C10 concrete was the fastest. The mean, median, and primary distribution ranges of both the thrust and torque are the smallest for the C10 specimen. The average thrust and torque also follow the same trend, increasing as the strength of the drilled medium increases.

In terms of the three-axis vibration data, the standard deviation of the vibration velocity along all three axes is the smallest in the C20 specimen. The mean vibration velocities in the y- and z-directions exhibit a decreasing trend as the strength of the drilled medium increases.

### 4.2. Comparative Analysis of Drilling Signals in Same-Strength Concretes

**Figure 16:**
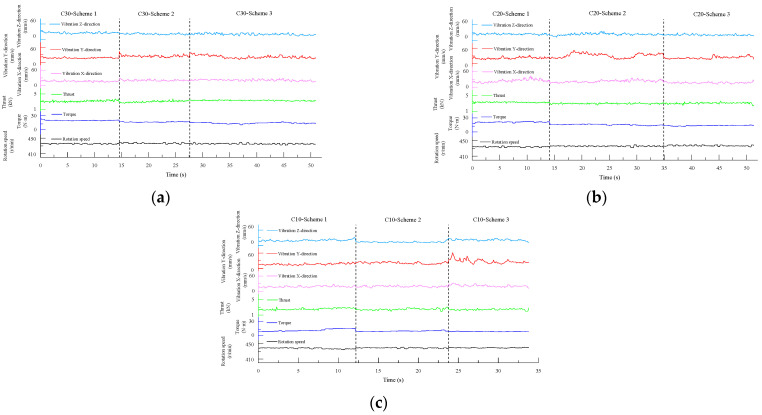
Concatenated drilling signals from specimens of the same strength across different groups. (**a**) Drilling signal combination for C30 strength. (**b**) Drilling signal combination for C20 strength. (**c**) Drilling signal combination for C10 strength.

To more comprehensively analyze and compare the drilling signal characteristics of the different-strength concretes during the drilling process, and to investigate whether the drilling signal parameters of the same-strength concretes in different experimental groups were consistent and reliable, the stable drilling signal data from each concrete specimen in the three experimental groups were concatenated, as shown in Figure 16.

Overall, although the signals from specimens of the same strength at different positions across the experimental groups were concatenated, the values of the drilling signal indicators remained largely consistent within each strength category.

**Figure 17:**
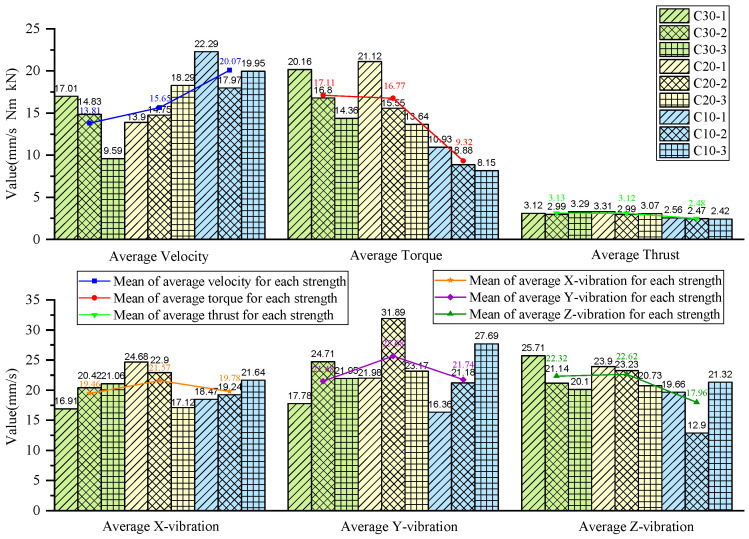
Statistical plot of average signal values across different experimental groups.

For further analysis, the average values of the drilling signals from the specimens with the same strength in each group were calculated, as shown in Figure 17.

The drilling speed in the lowest-strength C10 concrete is higher than those in the other two higher-strength concrete specimens. The average torque, average thrust, and average z-axis vibration values are all lower for the C10 concrete compared to the other two, which represent more stable rock layers. Notably, the differences in the drilling speed and average torque are significant. For the three-axis vibration velocity, the x- and y-directions are less effective at identifying the weakest drilled medium, while the z-axis vibration velocity in the C10 concrete is the smallest among the three specimens.

### 4.3. Analysis of Dimensionless Time-Domain Characteristics and Frequency-Domain Indicators of Drilling Signals

Taking the thrust, torque, and z-axis vibration velocity signal data from Experiment Scheme One as an example, statistical analysis was performed on the dimensionless time-domain indicators of the signals. The results are presented in Table 2.

The statistical results show that, among the dimensionless time-domain indicators and frequency-domain characteristics of the drilling signals, the pulse factor, peak factor, and margin factor for the three signals (thrust, torque, and z-axis vibration) are higher in the lowest-strength C10 concrete than in the other two specimens. However, the frequency characteristics, such as the center-of-gravity frequency and frequency standard deviation, are lower in the C10 concrete compared to the other specimens.

Despite these trends, the differences in some indicator values are small and not particularly distinct. Furthermore, other indicators show poor differentiation between concrete specimens of different strengths, making it challenging to rely solely on the dimensionless time-domain and frequency-domain characteristics of the signals for accurate identification.

Considering the data from all three experimental groups, the average values were calculated, with the results presented in Table 3. From the averaged data, it can be observed that in the lowest-strength C10 concrete, the centroid frequency of the thrust and torque signals is lower than that of the higher-strength specimens. Moreover, the waveform factor, impulse factor, peak factor, and margin factor of the torque, thrust, and z-axis vibration velocity signals are higher in the C10 concrete compared to the stronger specimens. However, the differences in the values of these dimensionless indicators are not particularly significant.

### 4.4. Analysis of Time–Frequency-Domain Characteristics of Signals

The Reassigned Short-Time Fourier Transform (RSTFT) [27,28,29,30] is an improved version of the Short-Time Fourier Transform (STFT) [31,32,33,34], which achieves higher time–frequency resolution by reassigning the power within the time–frequency spectrum to more precise time and frequency locations. The RSTFT first applies a standard STFT to the signal, generating an initial time–frequency spectrum. Then, by calculating the local phase derivatives at each time–frequency point, it determines more accurate time and frequency coordinates and reassigns the power at that point to these precise locations. This process concentrates the otherwise blurred power density, resulting in a clearer frequency distribution, significantly enhancing the resolution of the time–frequency spectrum and effectively reducing frequency blurring.

**Figure 18:**
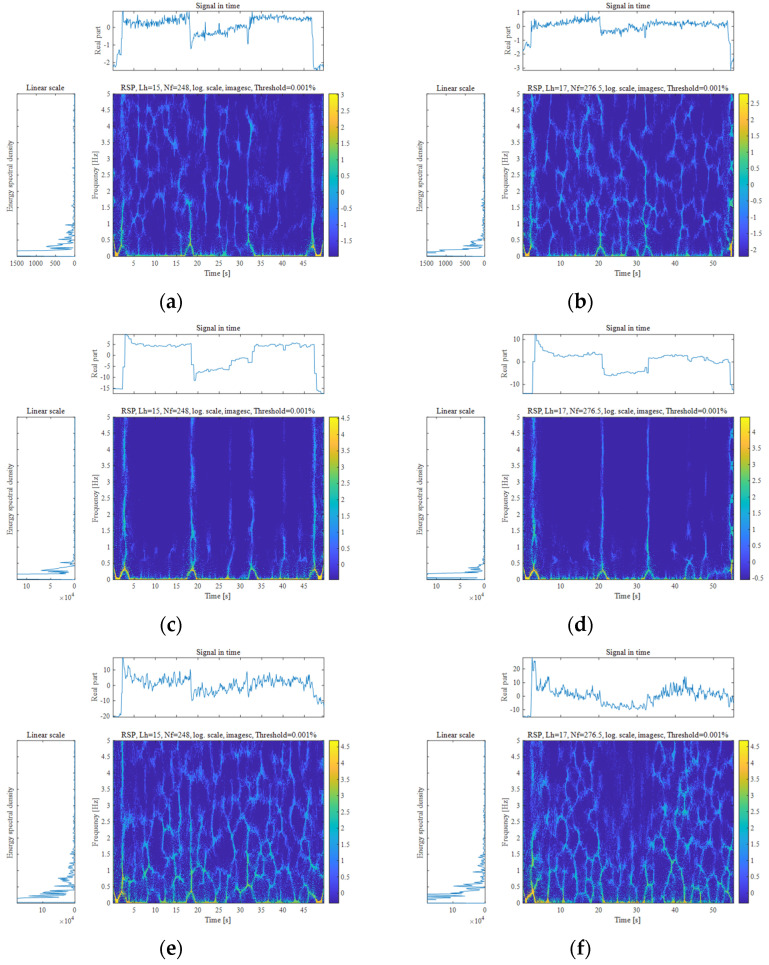
Time–frequency analysis of signals in Schemes 1 and 2. (**a**) Thrust signal in Scheme 1. (**b**) Thrust signal in Scheme 2. (**c**) Torque signal in Scheme 1. (**d**) Torque signal in Scheme 2. (**e**) Z-axis vibration velocity in Scheme 1. (**f**) Z-axis vibration velocity in Scheme 2.

Given that the vibration velocity data in the x- and y-directions show limited effectiveness at identifying weak media, the thrust, torque, and z-axis vibration velocity data from Experiment Groups 1 and 2 were subjected to RSTFT. The time–frequency-domain results are presented in Figure 18.

At the interfaces between different media, the torque signal exhibits distinct vertical lines spanning the entire frequency domain in the time–frequency representation, indicating clear signal characteristics. The thrust signal also displays vertical lines across multiple frequencies at these interfaces, though its features are less pronounced compared to the torque signal. In contrast, the time–frequency results of the z-axis vibration velocity data are more mixed, with unclear signal characteristics.

### 4.5. Analysis of Signal Characteristics Through Empirical Mode Decomposition

Empirical mode decomposition (EMD) [35,36,37] is a time–frequency analysis method designed for processing nonlinear and non-stationary signals. It adaptively decomposes complex signals into several simpler, physically meaningful sub-signals known as Intrinsic Mode Functions (IMFs). Each IMF represents a specific frequency component of the original signal, and these components are arranged in descending order of frequency. This decomposition allows for a detailed examination of the signal’s structure and can reveal underlying patterns that may not be apparent in the raw data.

**Figure 19:**
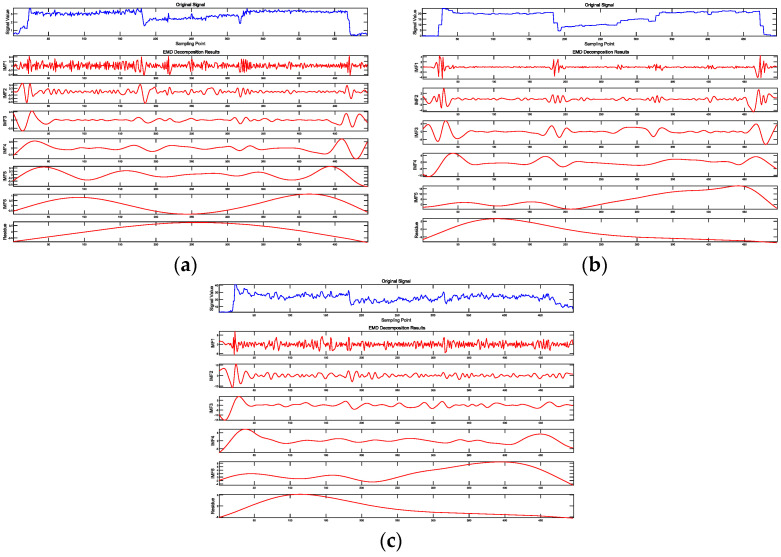
EMD results of drilling signals in Scheme 1. (**a**) EMD result of thrust signal. (**b**) EMD result of torque signal. (**c**) EMD result of z-axis vibration velocity signal.

**Figure A1:**
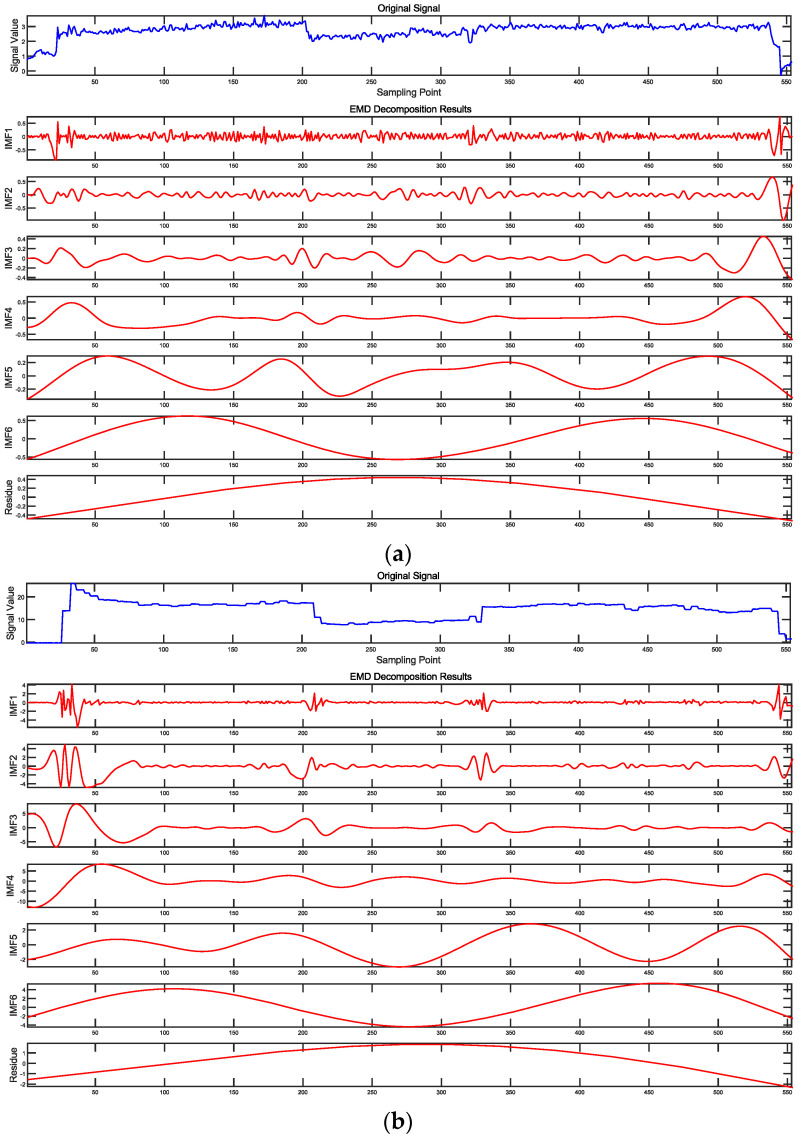

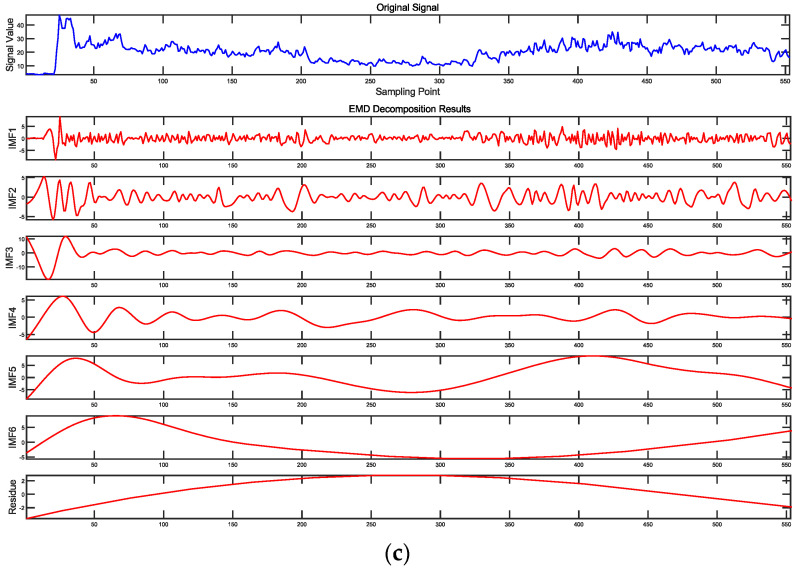
EMD results of drilling signals in Scheme 2. (**a**) EMD result of thrust signal. (**b**) EMD result of torque signal. (**c**) EMD result of z-axis vibration velocity signal.

**Figure A2:**
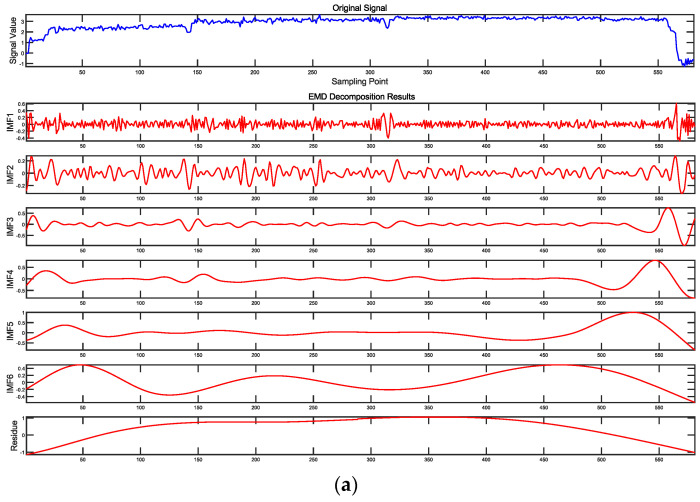

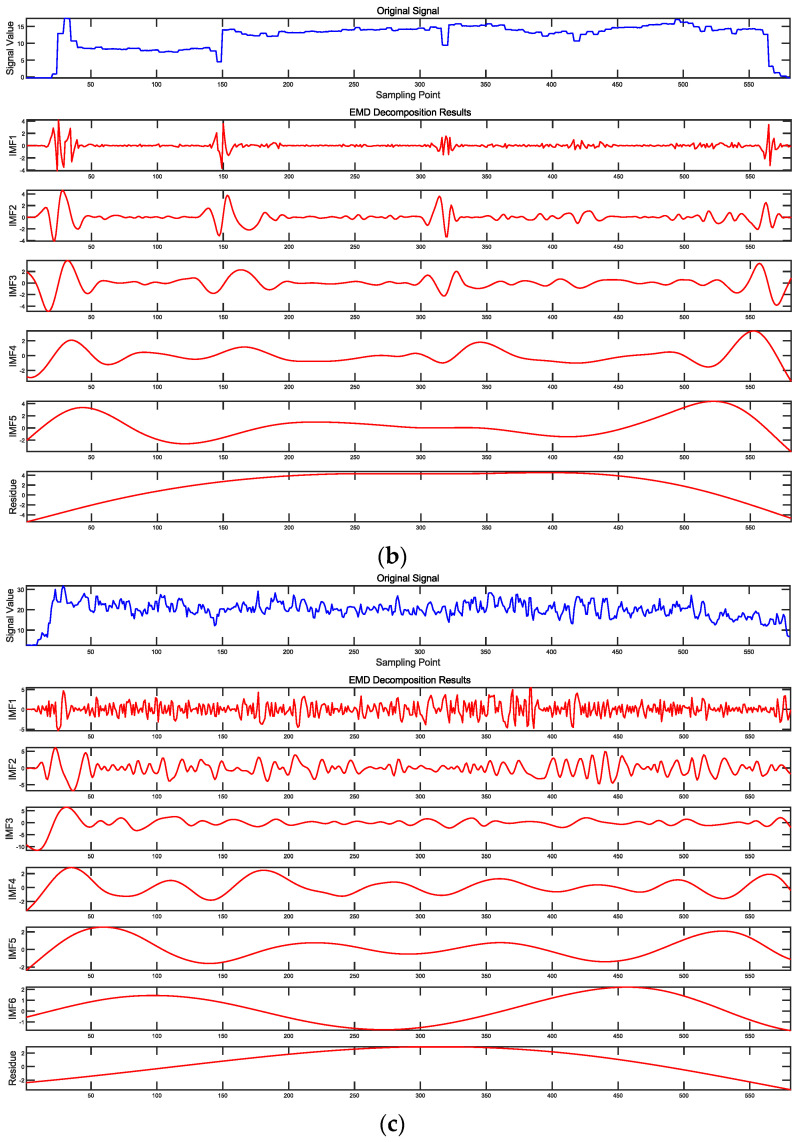
EMD results of drilling signals in Scheme 3. (**a**) EMD result of thrust signal. (**b**) EMD result of torque signal. (**c**) EMD result of z-axis vibration velocity signal.

The thrust, torque, and z-axis vibration velocity signal data from Experiment Scheme 1 were subjected to EMD. The decomposition results are shown in Figure 19. The EMD results of the other two experimental groups are shown in Figure A1 and Figure A2.

Significant changes in the signal are typically accompanied by corresponding variations in the IMFs derived from the EMD at the same positions. These signal fluctuations often occur at transitions between different drilling media. As mentioned earlier, the signals commonly exhibit a “V-shaped” pattern, with notable variations in the drilling signals. Correspondingly, the IMFs show pronounced oscillations at these points. Thus, this pattern of signal variation can serve as an auxiliary indicator for identifying the interfaces between different drilling media.

## 5. Discussion

### 5.1. Analysis of the Effect of Specimen Position on Drilling Signal Identification Results

As the drilling depth increases, the constraint effect of the borehole wall on the drill rod’s vibrations becomes more significant. Consequently, the vibration signals captured by the sensors are not solely generated by the rock-breaking action of the two-wing PDC bit; they also include vibrations resulting from interactions between the drill rod and the borehole wall.

As shown in the statistical results in Figure 17, for the C10 concrete, the specimen was positioned in the middle layer in the first two experiments, whereas in the third experiment, it was located at the bottom of the specimen stack and was the first to be drilled. During the initial contact between the drill bit and the C10 concrete specimen, substantial overall vibration was generated. As a result, the mean vibration velocities in the non-axial directions were higher than those observed when the C10 specimen was in the middle layer in the previous two experiments.

The previously described method demonstrated good effectiveness at identifying the weakest medium. However, its ability to distinguish between the other two higher-strength media was less effective compared to the identification of the weakest medium. When the strengths of the media are similar, differentiation becomes even more challenging. The greater the strength difference between drilling media, the more pronounced the differences in the signal indicators, resulting in better identification. Conversely, when the strengths are similar, the drilling signals tend to be alike, making precise and effective identification more difficult.

From Figure 6, Figure 7, Figure 10, Figure 11, Figure 14 and Figure 15, it can be observed that for the first two experimental groups, the C20 concrete placed at the top layer shows higher vibration velocity values and standard deviations in both the x- and y-directions compared to the other two specimens placed below. In the third experimental group, the C30 concrete at the top layer shows higher vibration velocity values and standard deviations in the x- and y-directions compared to the C30 specimen at the bottom of the previous two groups, while the C20 concrete in the middle layer shows lower vibration velocity values and standard deviations in the x- and y-directions. The reason for this may be that the deflection of the drill rod itself can cause vibrations in the x- and y-directions during the drilling process. Additionally, the drill rod may collide with the bottom boundary of the borehole and the borehole wall, and as the drilling depth increases, the frequency of collisions between the drill rod and the borehole wall may also increase, leading to higher vibrations in the x- and y-directions. These vibrations in the x- and y-directions are also captured by the vibration sensors. Furthermore, as the drilling depth increases, the height of the drilling rig rises, which reduces the distance between the vibration sensor and the specimen, bringing the sensor closer to the vibration source, potentially leading to higher recorded vibration velocities. The relatively stable vibration signals in the z-direction are mainly because this direction aligns with the drilling axis and is less influenced by other external factors.

From the above analysis, it can be concluded that the vibration velocity signals in the x- and y-directions may be influenced and disturbed by other factors, leading to instability, whereas the vibration velocity signal in the z-direction is relatively stable and reliable compared to the other two directions.

### 5.2. Integrated MWD Signal Features for Detecting Rock Strength Degradation Areas

From Figure 4, Figure 8 and Figure 12, it can be observed that for drilling near the interface between two different media, the thrust signal in the time domain exhibits a “V-shaped” distribution, characterized by an initial decrease followed by an increase. The experimental results indicate that the thrust and torque of the drill bit increase with the strength of the drilled medium, while structural weaknesses in the rock, such as joints and fractures, often reduce the rock strength, making the drilling process easier. From the experimental results presented earlier, it is evident that thrust signals are more sensitive to these strength degradation areas. Near interfaces between different media or in regions with small joints and fractures, the strength of the drilled medium decreases to varying degrees, resulting in fluctuations in the MWD signals. Monitoring fluctuations in these parameters allows MWD systems to effectively identify these geological structures [38]. This characteristic aligns with the findings in the studies [39,40], which reported a significant reduction in the thrust when drilling through fault zones. Such changes are reflected in the drilling signals, with abrupt signal variations especially noticeable at medium interfaces.

For the PDC drill bit contacting a new medium interface, the RSTFT time–frequency plots display impact signals spanning the entire frequency domain, with this characteristic being particularly pronounced in the time–frequency plots of the torque signals. This indicates that this method has strong potential for identifying medium interfaces. Additionally, the IMFs obtained from the EMD show significant fluctuations at the interfaces, which can be used as auxiliary indicators for distinguishing between different media.

Near the areas of rock strength degradation, the RSTFT time–frequency plots display impact signals spanning the entire frequency domain, with the torque signals being the most distinct. The first few IMFs from the EMD show significant fluctuations, and “V-shaped” distributions appear in the time-domain plots, with the thrust signals being the most prominent. These MWD signal features provide reliable evidence for identifying medium interfaces, small joints, fractures, faults, and other areas of rock strength degradation. Furthermore, these features can serve as prior knowledge for machine learning and deep learning models, aiding in the automated identification of these areas.

Therefore, the combined application of MWD signal features in the time domain, time–frequency domain, and EMD effectively detects subsurface rock changes as well as discontinuous geological structures such as joints and fractures. This has significant practical implications for preventing geological disasters like roof collapses in roadways and improving the safety and efficiency of the drilling process.

## 6. Conclusions

By using laboratory drilling experiments, a multi-parameter and multi-indicator analysis method involving the time domain, the time–frequency domain, and EMD was applied to study the response patterns of drilling signals as the PDC drill bit penetrated concrete media of varying strengths. The results showed that the average drilling speed effectively reflects the strength of the drilled medium; the lower the medium strength, the faster the drilling speed of the PDC bit. In weak media, the average thrust and torque indicators of the drilling signals were significantly lower than those in the higher-strength media. Regarding the three-axis vibration velocity indicators, the z-axis vibration velocity showed better differentiation for weak media compared to the x- and y-axes. The RSTFT and EMD methods can effectively identify the interfaces between two different media, minor joints, fractures, and other areas with medium changes or rock mass strength deterioration.

However, the dimensionless time-domain indicators and frequency-domain characteristics of the drilling signals show limited effectiveness at distinguishing weak media. Statistical analysis revealed that certain indicators, such as the pulse factor, peak factor, and margin factor, exhibited minimal differences in their values, making it difficult to effectively differentiate between media of varying strengths. This indicates that the current analysis methods for the dimensionless time-domain and frequency-domain characteristics of drilling signals have limitations in identifying weak rock formations, highlighting the need for further research and improvement.

## Figures and Tables

**Table 1:** Sensor specifications.

No.	Item	Parameter	Value
1	Acquisition device	Number of channels	16
		Range	0–20 mA
		Accuracy	<0.1%
		Mode	Continuous acquisition
2	Vibration sensor	Range	0~0.1 m/s
		Accuracy	2%
		Frequency response	10~1000 Hz
		Output current	4~20 mA
3	Distance sensor	Range	0~2 m
		Accuracy	0.05%
4	Thrust sensor	Range	0~8000 kg
		Accuracy	0.05%
5	Torque and rotational speed sensor	Maximum torque range	200 N m
		Accuracy	0.5%

**Table 2:** Dimensionless time-domain characteristics and frequency-domain indicators of drilling signals in Scheme 1.

	Torque			Thrust			Z-Axis Vibration Velocity		
	C30	C20	C10	C30	C20	C10	C30	C20	C10
Waveform Factor	1.001	1.001	1.027	1.002	1.000	1.004	1.006	1.004	1.013
Pulse Factor	1.183	1.063	1.386	1.177	1.107	1.206	1.344	1.245	1.552
Peak Factor	1.182	1.062	1.349	1.174	1.106	1.201	1.335	1.239	1.533
Margin Factor	1.183	1.063	1.404	1.178	1.107	1.208	1.348	1.247	1.562
Skewness	2.116	−1.562	0.596	0.435	−0.056	0.259	0.525	−0.124	0.703
Kurtosis	9.067	6.342	1.700	3.115	3.822	2.093	3.530	2.925	3.712
Center-of-Gravity Frequency	44.302	72.898	23.624	169.278	210.814	107.362	96.639	141.767	86.748
Frequency Standard Deviation	78.574	89.267	48.445	159.957	154.073	148.497	101.837	122.060	97.756

**Table 3:** Dimensionless time-domain indicators of drilling signals for concrete specimens with the same strength across different experimental schemes.

	Torque			Thrust			Z-Axis Vibration Velocity		
	C30	C20	C10	C30	C20	C10	C30	C20	C10
Waveform Factor	1.002	1.002	1.011	1.002	1.001	1.003	1.009	1.008	1.012
Pulse Factor	1.150	1.085	1.252	1.166	1.132	1.204	1.356	1.387	1.523
Peak Factor	1.148	1.084	1.238	1.164	1.131	1.200	1.344	1.376	1.505
Margin Factor	1.151	1.086	1.260	1.167	1.132	1.206	1.363	1.393	1.532
Skewness	0.755	−0.930	0.320	0.223	−0.407	0.012	0.417	0.323	0.808
Kurtosis	5.007	3.799	2.800	2.920	4.447	2.970	3.000	3.463	5.180
Center of Gravity Frequency	37.284	39.096	36.518	150.095	184.551	124.131	97.833	117.355	102.613
Frequency Standard Deviation	68.249	63.374	62.439	155.156	143.950	131.838	105.901	114.703	98.142

## Data Availability

The data that support the findings of this study are not publicly available due to the data being part of ongoing research but are available from the corresponding author upon reasonable request.
